# The quality characteristics and microbial communities of three components in traditional split‐fermented red sour soup

**DOI:** 10.1002/fsn3.4317

**Published:** 2024-07-19

**Authors:** Na Liu, Yue Hu, Mingxia Wu, Likang Qin, Aiming Bao, Weijun Qin, Song Miao

**Affiliations:** ^1^ School of Liquor and Food Engineering Guizhou University Guiyang China; ^2^ Chongqing Jiangjin Grain Reserves Co., Ltd Chongqing China; ^3^ Guizhou Nanshanpo Food Processing Co., Ltd Anshun China; ^4^ Teagasc Food Research Centre, Moorepark Fermoy, Co.Cork Ireland

**Keywords:** flavor, microbial community, quality, spilt‐fermented red sour soup

## Abstract

Red sour soup is a Guizhou specialty condiment made by the natural fermentation of tomatoes and chili. In this study, three components (tomato acid, chili acid, and tomato and chili mixed acid) of split‐fermented red sour soup were explored to compare the quality characteristics and microbial communities in the middle and late fermentation stages. The titratable acidity of mixed acids was lower than that of tomato acid and chili acid in the middle stage, but it was significantly increased in the late stage. The cell viability of lactic acid bacteria was mostly higher than that of yeasts during the whole fermentation. Also significantly increased in the late stage of fermentation were sensory scores and the signal intensity of sour substances. However, the signal intensity of both bitter and salty substances decreased, and the total amount of free amino acids was reduced. In addition, the antioxidant capacity of the samples and the dominant microorganisms were different between the two fermentation stages, *Lactobacillus* and *Kazachstania* were the key common genus of the different components of split‐fermented red sour soup. It is anticipated that this study would provide us an insight into the quality characteristics and microbial communities of split‐fermented red sour soup.

## Graphical abstract

In this study, three components (tomato acid, chili acid, and tomato and chili mixed acid) of traditional split‐fermented red sour soup were explored to compare the quality characteristics and microbial communities in the middle and late fermentation stages.

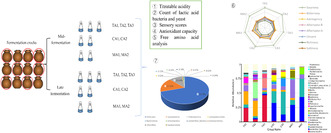

## 1 INTRODUCTION

Fermentation is an ancient way of food preservation and the preferred traditional process for preserving foods and enhancing their properties because it can effectively extend the shelf life of foods through the production of inhibitory metabolites such as organic acids, ethanols, and bacteriocins by microorganisms (Yao et al., 2022). Fermented foods have a long history and are an indispensable part of our daily life (Sun et al., 2021). Compared to unfermented foods, fermented foods are endowed with various properties, such as weight control, risk reduction of colorectal cancer, and improvement in gastrointestinal symptoms, which help to eliminate pathogens and strengthen the integrity of the intestinal barrier (Kocot & Wróblewska, 2021; Wastyk et al., 2021; Zhang et al., 2021). A wide variety of traditional fermented foods have existed in China for a long time. Based on raw materials, they can be mainly classified into wine foods, cereal foods, bean foods, vegetable foods, fruit foods, meat foods, aquatic foods, dairy products, and other foods (Chen et al., 2021). Fermented foods are widely accepted due to their unique flavor and rich nutrition. However, most fermented foods are produced by uncontrolled, spontaneous fermentation. It is crucial to understand and control the spontaneous fermentation of foods.

As a traditional naturally fermented condiment of the Miao and Dong Nations of Guizhou Province, China, sour soup is commonly used in the preparation of foods such as hot pots and stews, and can regulate intestinal flora and promote digestion (Li et al., 2021; Liu et al., 2021). Based on the raw materials used in the fermentation process, Guizhou sour soup is divided into two major categories: red sour soup and white sour soup. Different from white sour soup, red sour soup is prepared with tomatoes and chili through fermentation and further classified into split‐fermented red sour soup and mixed‐fermented red sour soup according to the preparation process (Lin et al., 2020; Wang et al., 2020).

Red sour soup is rich in nutrition and unique in color and flavor. Red sour soup contains a variety of necessary mineral elements of the human body, such as calcium, magnesium, phosphorus, sodium, and potassium, which are important for maintaining the excitability of nerves and muscles as well as acid–base balance (Pan et al., 2020). It is composed of various organic acids and functional ingredients and has the unique functions of seasoning, aroma enhancement, removal of fish and stink odors, grease relief, appetizer, anti‐fatigue, anti‐aging, immune regulation, and relieving cardiovascular diseases (Zheng et al., 2020). Moreover, it can prevent hyperlipidemia and reduce the fatty liver caused by a high‐fat diet (Cong et al., 2021; Yang et al., 2021). Sour soup is fermented in a relatively uncontrolled manner, and various microorganisms in the fermentation environment lead to the complex microbial community structure in sour soup (Cai et al., 2021; Lin et al., 2020). The metabolic activities of certain microbiota during fermentation contribute to the health function of fermented foods (Das et al., 2020). However, the main flora of traditional fermented vegetables varies with raw material, geography, climate, and manufacturing process. Although traditional fermentation techniques have been technically developed, the fermentation method of sour soup is spontaneous fermentation with many problems, such as microbial contamination.

Traditional red sour soup has a long fermentation period, ranging from 1 month to 12 months. The most common traditional fermentation process is to ferment fresh red chilies and tomatoes with glutinous rice, white wine, salt, garlic, etc., and then make semi‐finished products, and then mix the semi‐finished products of the primary fermentation, “Tomato sour soup” and “Chili sour soup,” according to a certain proportion to continue fermentation, then make delicious red sour soup after secondary fermentation, this process is called split‐fermented red sour soup. Another process is to directly ferment tomatoes, chilies, white wine, glutinous rice, salt, and others, this process only requires one round of fermentation, it is called mix‐fermented red sour soup. It is worth noting that, because of the split‐fermented red sour soup with separate materials and then mixed‐material fermentation, microbial community species are involved in the whole fermentation process, and the complex microbial community interactions prompted the red sour soup to produce organic acids, amino acids, and other flavor components, and to enhance the flavor quality of the red sour soup. Therefore, split‐fermented red sour soup is welcomed by consumers and widely used in the processing of “sour soup fish,” “sour soup beef,” hot pot seasoning, and other food products, which has a broad market demand prospect. However, there was a lack of research on split‐fermentation.

This study aims to analyze and compare the physicochemical indexes and microbial composition of the different components of red sour soup (tomato acid, chili acid, and mixed acid) in the middle and late fermentation stages through physicochemical experiments and analysis techniques. Including electronic tongue detection, E‐tongue is a digital assessment technique of sensory indicators of food products based on artificial lipid membrane technology and has the advantages of high accuracy and reproducibility (Cai et al., 2021). The study provides a theoretical basis for the development of suitable fermentation agents for red sour soup.

## 2 MATERIALS AND METHODS

### 2.1 Materials

Hydroxyl radical, DPPH, and ABTS free radical scavenging ability test kits were purchased from Beijing Solarbio Life Sciences Co., Ltd., China. Phenolphthalein indicator, pure water. anhydrous ethanol, sodium hydroxide, hydrochloric acid, sulfosalicylic acid, and so on were all domestic analytical pure.

### 2.2 Sample

**FIGURE 1:**
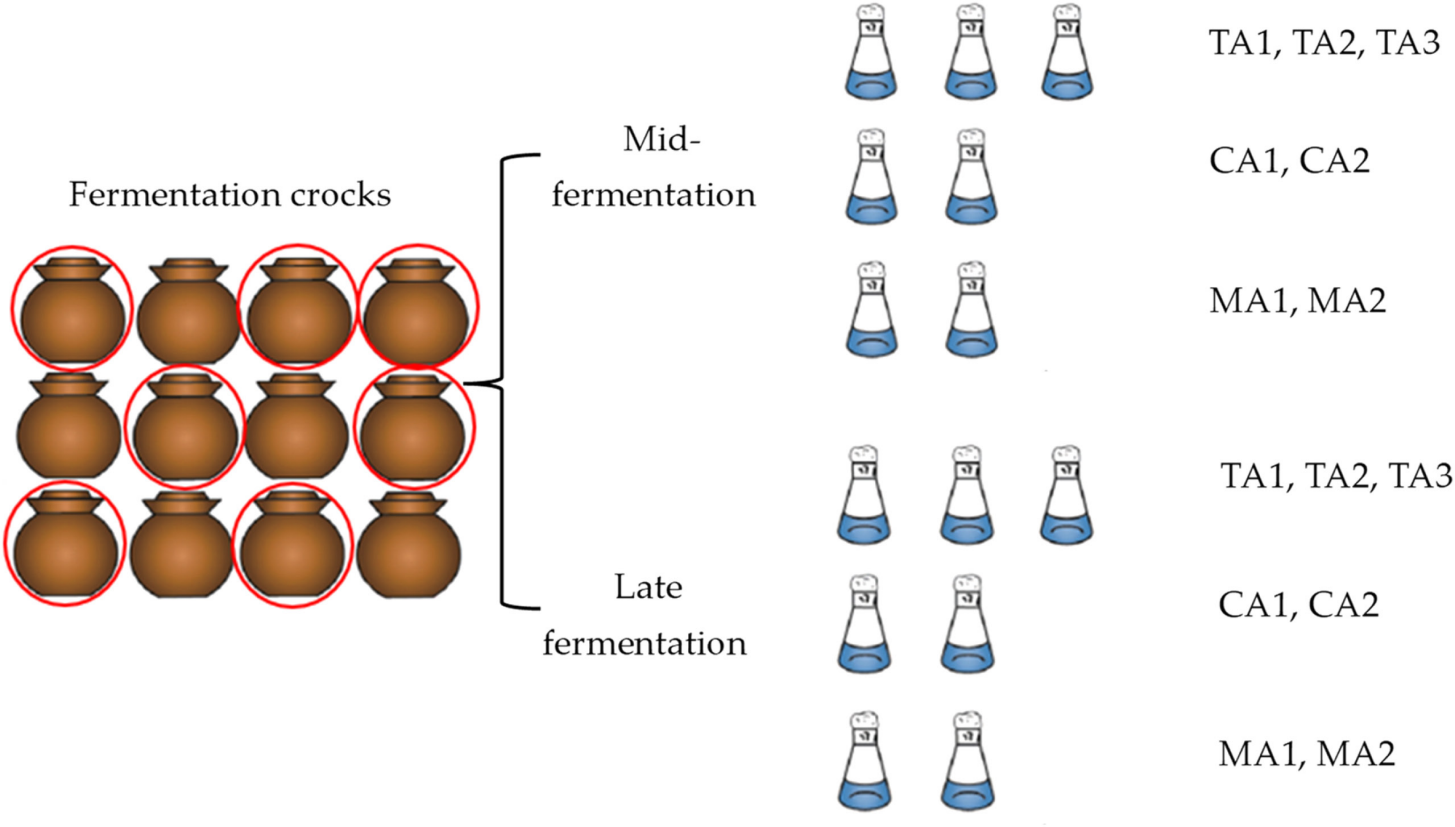
The diagram of a sampling of traditional split‐fermented red sour soup.

All the samples mainly included three components (Figure 1): tomato acid, chili acid, and mixed acid of the different parts of red sour soup in the middle and late fermentation stages, and the samples were produced by Guizhou Nanshanpo Food Processing Co., Ltd. A total of seven samples were respectively collected in November 2021 (middle fermentation stage) and July 2022 (late fermentation stage), including three kinds of tomato acid (TA1, TA2, and TA3), two kinds of chili acid (CA1 and CA2), and two kinds of mixed acid (MA1 and MA2). All samples are transported to the laboratory under sterile (aseptic bags) and 4°C conditions. The pre‐treatment of raw materials and the fermentation environment for the same batch of samples from the same company were the same, the products showed no significant difference in the early fermentation stage. Therefore, no sample was collected in the early fermentation stage.

### 2.3 Determination of titratable acidity

Titratable acidity was determined according to the previous method with minor modifications (Zheng et al., 2022). Firstly, 5.0 g of the sample was weighed in a beaker and washed several times with CO_2_‐free water into a 100 mL volumetric flask, in which water was added to the volume of 100 mL and mixed well. Then, 20 mL of the diluted sample was accurately transferred into another beaker. After adding 1–2 drops of phenolphthalein indicator and mixing well, the mixture solution was titrated with sodium hydroxide standard solution [c(NaOH) = 0.0501 mol/L] under rotation conditions for complete mixing until phenolphthalein turned red and the color of the reaction solution did not change for 30 s. Then, the number of milliliters of sodium hydroxide standard titration solution consumed (*V*_1_) was recorded for subsequent calculation. A blank experiment was also performed under the same conditions, except that the 20‐mL diluted sample was replaced with the corresponding volume of water. The volume of sodium hydroxide standard titration solution consumed (*V*_0_) was also recorded. The titratable acidity is calculated as:

(1) $$X=C\times \left(V_{1}-V_{0}\right)\times K\times F\times 1000/M$$

where *C* indicates the concentration of the standard titration solution of sodium hydroxide (mol/L); *V*_1_ indicates the volume of standard titration solution of sodium hydroxide consumed for titration (mL); *V*_0_ indicates the volume of standard titration solution of sodium hydroxide consumed by blank (mL); *K* indicates the acid conversion factor (for lactic acid, *K* = 0.090); *F* is the dilution times; *M* is the sample mass (g).

### 2.4 Counting of lactic acid bacteria and yeasts

Lactic acid bacteria and yeasts in samples were counted according to the previous method with minor modifications (Zheng et al., 2022). First, a 10 g sample was accurately weighed into 90 mL of 0.8% saline in a sterile environment, and then a 10‐fold serial dilution was performed. According to the estimation results of the total number of viable bacteria in the samples to be examined, two to three serial dilutions were selected, and 200 μL of each dilution was evenly pasted on sterilized dishes of MC, MRS, and Bengal Red Agar media. Two parallel dishes were used for each dilution. The inoculated MC dishes were incubated aerobically at 36 ± 1°C for 72 ± 2 h. MRS agar dishes were incubated anaerobically at 36 ± 1°C for 72 ± 2 h, and Bengal red agar cultures were incubated aerobically at 28 ± 1°C for 5 d for counting.

### 2.5 Sensory evaluation

The sensory panel consisted of 10 members of the food discipline who often consumed red sour soup. The scoring items and scores were slightly modified from the previous study (Yang et al., 2021). Sensory evaluators were trained to understand and be familiar with the sensory characteristics of sour soup quality, such as color, scent, texture, and palate. The members rinsed their mouths with purified water before sensory evaluation. The samples were randomly coded, and the sensory evaluation of the samples was conducted in terms of color, odor, texture, and taste (Table 1).

**TABLE 1:** Sensory evaluation standard of samples.

Evaluation indexes	Scores	Evaluation criteria and scores	
Color	10	Bright red, shiny	8.1–10.0
		Red, shiny	6.1–8.0
		Orange red with a slight luster	4.1–6.0
		Pale yellow, less shiny	2.1–4.0
		Almost colorless and lusterless	0.1–2.0
Scent	10	Rich fragrance, harmonious smell, odorless	8.1–10.0
		Good aroma, harmonious smell, and odorless	6.1–8.0
		Lighter fragrance, slightly harmonious smell, and odorless	4.1–6.0
		Very light fragrance, not very harmonious smell, and odorless	2.1–4.0
		Fragranceless and odorless	0.1–2.0
Texture	10	Uniform sauce, uniform texture, moderately thick and delicate	8.1–10.0
		Uniform sauce, uniform texture, no sediment, suspended matter	6.1–8.0
		Relatively uniform sauce, uniform texture, slight precipitation, suspension	4.1–6.0
		Slightly dispersed sauce, with sedimentation and suspension	2.1–4.0
		Dispersed sauce with sediment and suspended solids	0.1–2.0
Palate	10	Rich taste, suitable saltiness, strong aftertaste, no peculiar taste	8.1–10.0
		Good taste, suitable saltiness, strong aftertaste, no peculiar smell	6.1–8.0
		Slightly better texture, suitable for salty taste, good aftertaste, no peculiar taste	4.1–6.0
		Light palate, slightly bitter, slightly peculiar	2.1–4.0
		Very light taste, obvious bitter taste, with an odor	0.1–2.0

### 2.6 Determination of taste substances

Taste substances in samples were determined according to the previous method (Liu et al., 2021). First, 25 g of sample was diluted to a volume of 250 mL and then the clarified solution was taken after filtration for detection with an electronic tongue taste analysis system (Insent SA‐402B, Atsugi‐chi, Japan). The same sample was determined four times. The first experimental data were removed, and the average of the other three data points was taken for the taste substance analysis.

### 2.7 Determination of antioxidant properties

#### 2.7.1 Determination of hydroxyl radical scavenging ability

The hydroxyl radical scavenging capacity of samples was determined with the hydroxyl radical scavenging capacity assay kit (Beijing Solarbio Science & Technology Co., Ltd.). Firstly, 0.1 g of the sample was weighed, then transferred into 1 mL of the extraction solution for ice bath homogenization, and centrifuged at 10,000 r/min for 10 min at 4°C. Then, the supernatant was obtained, and reagents were added according to the instructions of the kit, vortexed, and mixed well for a water bath at 37°C for 60 min. Then, the reaction mixture was centrifuged at 10,000 r/min for 10 min at room temperature. The wavelength was adjusted to 536 nm, and the instrument was zeroed with distilled water. The absorbances at A_536 nm_ of the supernatants of blank, control, and experimental samples were measured and recorded as *A*_blank_, *A*_control_, and *A*_measurement_, respectively. The hydroxyl radical scavenging capacity (*D*%) is calculated as:

(2) $$D\%=A_{\text{measurement}}-A_{\text{control}}/A_{\text{blank}}-A_{\text{control}}\times 100\%$$

#### 2.7.2 Determination of DPPH free radical scavenging capacity

The DPPH radical scavenging capacity was determined with the DPPH radical scavenging capacity assay kit (Beijing Solarbio Science & Technology Co., Ltd.). The samples were first dried in an oven at 60°C until a constant weight and passed through a 40‐mesh sieve in a mortar. Then, 0.05 g of the sieved sample was weighed and transferred into 1 mL of extraction solution, which was then put in a water bath at 40°C for 30 min. The mixture was centrifuged at 10,000 r/min for 10 min at room temperature. Then, the supernatant was obtained, and the reagents were added according to the instructions of the kit, vortexed, and mixed well. The reaction solution stood for 30 min at room temperature in the dark to avoid light. The absorbances at *A*_515 nm_ of the supernatants of blank, control, and experimental samples were measured and recorded as *A*_blank_, *A*_control_, and *A*_measurement_, respectively. The DPPH radical scavenging capacity (*D*%) is calculated as:

(3) $$D\%=A_{\text{blank}}-\left(A_{\text{measurement}}-A_{\text{control}}\right)/A_{\text{blank}}\times 100\%$$

#### 2.7.3 Determination of ABTS free radical scavenging capacity

The ABTS free radical scavenging capacity was determined with the ABTS free radical scavenging capacity assay kit (Beijing Solarbio Science & Technology Co., Ltd.). The samples were first dried in an oven at 60°C until a constant weight was reached and passed through a 40‐mesh sieve in a mortar. Then, 0.05 g of the sieved sample was weighed and transferred into 1 mL of extraction solution, which was then put in a water bath at 40°C for 30 min. The mixture was centrifuged at 10,000 r/min for 10 min at room temperature. Then, the supernatant was obtained, and the reagents were added according to the instructions of the kit, vortexed, and mixed well. The reaction solution stood for 6 min at room temperature in the dark to avoid light. The absorbances at *A*_405 nm_ of the supernatants of blank, control, and experimental samples were measured and recorded as *A*_blank_, *A*_control_, and *A*_measurement_, respectively. The ABTS free radical scavenging capacity (*D*%) is calculated as:

(4) $$D\%=A_{\text{blank}}-\left(A_{\text{measurement}}-A_{\text{control}}\right)/A_{\text{blank}}\times 100\%$$

### 2.8 Determination of free amino acids

The sample solution for determining free amino acids was prepared according to the following steps. First, 0.5 g of sample was accurately weighed, ground, dissolved in 0.01 mol/L HCl, diluted to a volume of 50 mL with 0.01 mol/L HCl, sonicated for 30 min, shaken, and filtered. Then, 2 mL of the filtrate was accurately transferred into a centrifuge tube, and 2 mL of 8% sulfosalicylic acid was added and mixed well. After standing for 15 min, the mixture was centrifuged at 10,000 r/min for 10 min. Then, the supernatant was filtered through a membrane with a pore size of 0.45 μm and applied to the automatic amino acid analyzer (S‐433D; Hitachi, Ltd.) to determine the contents of free amino acids in the samples.

The determination conditions were set as follows: Wako 013–08393 amino acid mixture standard solution (2.5 μmol/mL type H diluted to 100 μmol/L), LCAK07/Li standard cationic exchange resin, injection volume of 50 μL, the detection wavelength of Channel 1 at 440 nm for the detection of proline, the detection wavelength of Channel 2 for the detection of other amino acids; infusion pump pressure of 3–4.2 MPa, the flow rate of the elution pump (0.45 mL/min), the flow rate of ninhydrin derivatization pump (0.25 mL/min), on‐line derivatization method, temperature of separation column (37°C), reactor temperature of 130°C, Mobile Phase A (citric acid‐lithium citrate buffer solution at pH 2.90), Mobile Phase B (citric acid‐lithium citrate buffer solution at pH 4.20), Mobile Phase C (citric acid‐lithium citrate buffer solution at pH 8.00), and Mobile Phase D (0.5 mol/L lithium hydroxide regeneration solution).

### 2.9 Illumina sequencing

It was tested by Novogene. The genomic DNA was first extracted with the CTAB method, and then the purity and concentration of DNA were checked by agarose gel electrophoresis. Then, an appropriate quantity of sample DNA was transferred into a centrifuge tube and diluted to 1 ng/μL with sterile water. Phusion® High‐Fidelity PCR Master Mix with GC Buffer and the high‐fidelity enzyme from New England Biolabs were used for PCR with different primers to ensure amplification efficiency and accuracy. 16S V4 region primers (515F and 806R) were used to identify bacterial diversity in the sample, and ITS1 region primers (ITS5‐1737F and ITS2‐2043R) were used to identify fungal diversity in the sample.

### 2.10 Statistical analysis

The physical and chemical analyses and E‐tongue measurements were performed in triplicate, and the measured data were expressed as mean ± standard deviation. The data were analyzed with SPSS Statistics 26.0 software to test the significant differences among means at *p* < 0.05.

## 3 RESULTS AND DISCUSSION

### 3.1 Titratable acidity and count of lactic acid bacteria and yeast

Titratable acidity (TA) is one of the key indicators of fermented vegetables and affects the growth of microorganisms, the accumulation of metabolites, and the fermentation state of final products. The total acidity values were significantly different among the seven samples in the middle fermentation stage and the late fermentation stage (*p* <0 .05, Table 2). Lactic acid bacteria decomposed carbohydrates in foods to produce lactic acid, so the titratable acidity increased with the increase in the yield of lactic acid (Gong et al., 2021). TA2 and CA2 had the highest total acidity (19.10 ± 0.23 g/kg and 19.64 ± 0.16 g/kg) in the middle fermentation. Some strains produced organic acids and increased the acidity, sugar metabolism by lactic acid bacteria also increased acidity (Peyer et al., 2016). Therefore, the difference in total acidity among various samples might be interpreted as follows. Some flora from the fermentation feedstock in the middle fermentation stage in the mixed acid samples inhibited each other, so the flora produced the lower levels of acidic components. The total acidity values of tomato acid samples or chili acid samples in the late fermentation stage were slightly lower than those in the middle fermentation stages because a single material could not provide enough substances required for the fermentation in the late stage and some products might be consumed. For example, acid‐tolerant microorganisms (*Staphylococcus*, *Lactiplantibacillus*, etc.) might oxidize lactic acid into acetic acid when the growth of lactic acid bacteria was limited by the increased level of lactic acid (Gong et al., 2021). Differently, the total acidity values of the mixed acid samples (MA1 and MA2) in the late fermentation stage were significantly higher than those in the middle fermentation stage because the different ingredients in the samples of mixed acids could provide multiple nutrients to the microorganisms in the fermentation system in the late fermentation stage and significantly increase acidic components.

**TABLE 2:** TA (g/kg) of the samples in the middle and late fermentation stages.

Samples	Middle fermentation TA (g/kg)	Late fermentation TA (g/kg)
TA1	13.44 ± 0.13^d^	13.52 ± 0.13^e^
TA2	19.10 ± 0.23^b^	18.88 ± 0.03^b^
TA3	8.57 ± 0.21^g^	7.74 ± 0.13^g^
CA1	17.86 ± 0.05^c^	17.71 ± 0.16^c^
CA2	19.64 ± 0.16^a^	19.30 ± 0.13^a^
MA1	10.65 ± 0.29^e^	15.28 ± 2.69^d^
MA2	9.72 ± 0.31^f^	11.47 ± 0.00^f^

*Note*: Data are expressed as mean ± standard deviation (three replicates). ^a–g^Different superscript letters in the same column indicate significant differences (*p* < .05).

Lactic acid bacteria generally possessed higher cell viability than yeasts in the middle and late fermentation stages (Table 3) because the closed fermentation environment of red sour soup contained a low content of oxygen and was more suitable for the growth of lactic acid bacteria. In addition, yeasts could possibly provide nutrients for lactic acid bacteria. In all seven samples except TA2, CA1, and CA2, the cell viability of lactic acid bacteria in the late fermentation stage was higher than that in the middle fermentation stage because limited fermentation substrates and accumulated organic acids decreased the cell viability of some lactic acid bacteria, which were less tolerant to acids (Nsogning Dongmo et al., 2016). Except that in CA1 and CA2, the cell viability of yeasts was enhanced, the cell viability of yeasts was reduced in all samples because the tolerance of most fungi to acids was lower than that of lactic acid bacteria, and the survival of yeasts was limited because some samples in the late fermentation stage could not provide sufficient carbon and nitrogen sources (An et al., 2021).

**TABLE 3:** Total numbers of lactic acid bacteria and yeasts (CFU/g) in the samples in the middle and late fermentation stages.

Samples	Middle fermentation lactic acid bacteria (CFU/g)	Late fermentation lactic acid bacteria (CFU/g)	Middle fermentation yeasts (CFU/g)	Late fermentation yeasts (CFU/g)
TA1	1.03 × 10^6^	1.72 × 10^6^	0.71 × 10^4^	0.10 × 10^4^
TA2	1.21 × 10^6^	0.51 × 10^6^	1.05 × 10^5^	0.10 × 10^5^
TA3	4.40 × 10^6^	4.90 × 10^6^	3.68 × 10^5^	0.05 × 10^4^
CA1	0.75 × 10^6^	0.10 × 10^6^	0.10 × 10^2^	1.90 × 10^4^
CA2	0.57 × 10^6^	0.43 × 10^6^	0.50 × 10^3^	6.50 × 10^3^
MA1	0.06 × 10^5^	0.65 × 10^5^	1.83 × 10^4^	1.40 × 10^4^
MA2	3.30 × 10^6^	5.02 × 10^6^	2.83 × 10^6^	0.14 × 10^6^

### 3.2 Sensory scores and taste substance analysis

CA1 and CA2 had higher sensory scores in the middle fermentation stage, whereas the sensory scores of the samples of tomato acid (TA1, TA2, and TA3) were generally lower (Table 4), indicating that the chili acid samples (CA1 and CA2) contained more abundant substances. In addition, the sensory scores of all samples in the late fermentation stage were significantly higher than those in the middle fermentation stage, and the samples of CA2 and MA2 had the highest sensory scores. During food fermentation, *Lactobacillus*, *Pichia*, and *Schizosaccharomyces* were the main contributors to the formation of flavor compounds (Wu et al., 2021). Lactic acid bacteria could decompose raw materials into flavor compounds such as amino acids, esters, and organic acids, which could enhance the flavor and nutrition of fermented foods (Cai et al., 2021) and also produce extracellular polysaccharides (EPS), which could improve the taste, texture, and stability of fermented foods (Belleggia et al., 2022). The sensory scores of late fermentation were increased, the reason might be that flavor and taste substances were increased after fermentation. Sensory scores showed significant differences among the seven samples at late fermentation (*p* < 0.05).

**TABLE 4:** Sensory scores of all the samples in the middle and late fermentation stages.

Samples	Middle fermentation scores	Late fermentation scores
TA1	24.76 ± 0.04^e^	29.27 ± 0.03^f^
TA2	25.13 ± 0.07^d^	31.4 ± 0.04^e^
TA3	22.22 ± 0.04^f^	28.97 ± 0.07^g^
CA1	31.01 ± 0.34^a^	33.38 ± 0.05^d^
CA2	29.57 ± 0.03^b^	36.49 ± 0.01^a^
MA1	25.61 ± 0.24^c^	33.68 ± 0.02^c^
MA2	29.34 ± 0.06^b^	35.5 ± 0.03^b^

*Note*: Data are expressed as mean ± standard deviation (three replicates). ^a–g^Different superscript letters in the same column indicate significant differences (*p*< .05).

**FIGURE 2:**
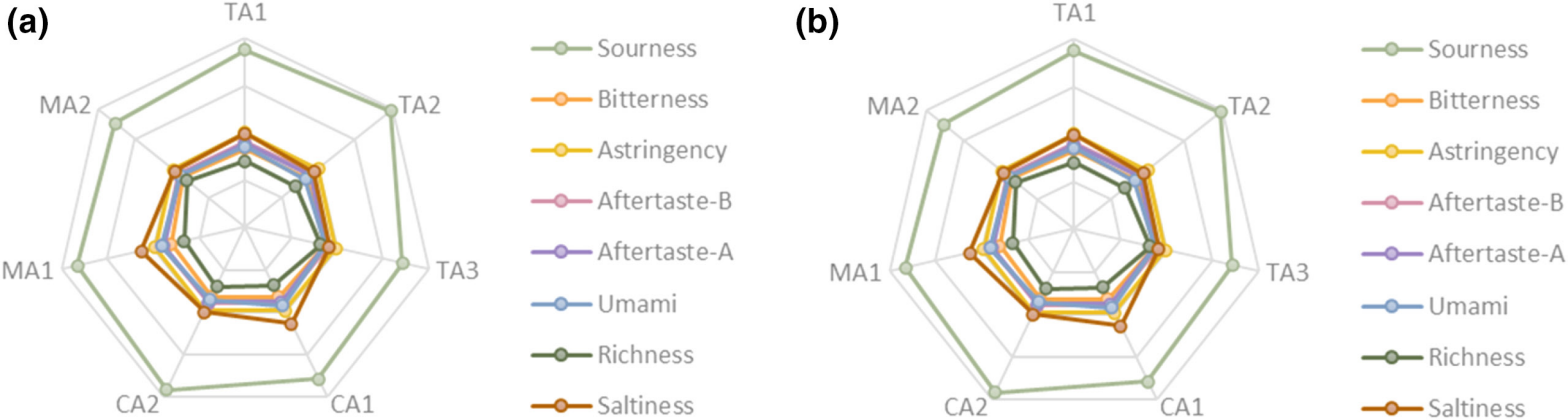
Taste signal radar chart of samples in (a) the middle fermentation stage and (b) the late fermentation stage.

The signal intensities of taste substances detected by the electronic tongue were different among different samples, and the signal intensities of taste substances in the samples were different between the middle and late fermentation stages (Figure 2). In the middle fermentation stage, the signal intensity of each taste substance showed no significant difference. In the late fermentation stage, sourness had the strongest flavor signal, and the intensity of bitter and salty signals decreased. TA3 had the lowest sour signal intensity and salty signal intensity, but had the highest bitter signal intensity. In the sensory evaluation, the score of TA3 was the lowest and corresponded to the highest bitter signal intensity.

### 3.3 Analysis of antioxidant capacity

Fermentation microorganisms have been reported to endow fermented products with enhanced antioxidant activity (Diez‐Ozaeta & Astiazaran, 2022). The interaction between yeasts and lactic acid bacteria can improve the antioxidant activity of foods (Rai et al., 2019). Yeasts could produce a variety of antioxidant compounds (Sadeghi et al., 2022), and EPS produced by lactic acid bacteria also had antioxidant activity (Belleggia et al., 2022). A study on fermented sausages showed that some free amino acids (FAAs) also exhibited strong antioxidant capacity and could scavenge free radicals (Luan et al., 2021). The highest scavenging rate of ABTS radicals and the low scavenging rate of DPPH radicals and OH^−^ radicals were found in all samples (Table 5). The OH^−^ radical scavenging rate of all samples in the late fermentation stage was significantly higher than that in the middle fermentation stage, and the difference in the OH^−^ radical scavenging rate was significant between the samples (*p* < .05). The changes in DPPH radical scavenging rate in most samples in the middle and late fermentation stages were not large, but the DPPH radical scavenging rates in CA2, MA1, and MA2 in the late fermentation were respectively about 1.94, 6.51, and 0.51 times higher than those in the middle fermentation stage because these samples significantly increased the cell viability of yeasts and lactic acid bacteria in the late fermentation stage and improved the antioxidant activity. The DPPH radical scavenging rate was significantly different among samples (*p* < 0.05) in both the middle and late fermentation stages. The ABTS radical scavenging rate of each sample was relatively stable in the middle and late fermentation stages and ranged from 92.58% to 99.46% in the middle fermentation stage and from 91.37% to 97.28% in the late fermentation stage. These samples showed different antioxidant capacity in two stages because different metabolites of lactic acid bacteria and yeasts and free amino acids were produced in different fermentation stages.

**TABLE 5:** Scavenging capacity of OH–, DPPH, and ABTS of samples in the middle and late fermentation stages.

Samples	Scavenging capacity of OH^−^ (%)	Scavenging capacity of DPPH (%)	Scavenging capacity of ABTS (%)
Middle fermentation			
TA1	0.94 ± 0.00^c^	6.45 ± 0.08^f^	96.26 ± 0.09^c^
TA2	0.38 ± 0.16^d^	37.3 ± 0.16^b^	97.11 ± 0.00^b^
TA3	4.14 ± 0.16^a^	20.66 ± 0.08^e^	92.58 ± 0.05^e^
CA1	0.87 ± 0.51^c^	25.63 ± 0.04^d^	96.23 ± 0.05^c^
CA2	1.25 ± 0.11^c^	28.91 ± 0.05^c^	96.21 ± 0.05^c^
MA1	2.54 ± 0.06^b^	3.28 ± 0.12^g^	94.14 ± 0.00^d^
MA2	0.00 ± 0.00^e^	61.67 ± 0.08^a^	99.46 ± 0.05^a^
Late fermentation			
TA1	10.23 ± 0.00^d^	7.6 ± 0.08^g^	91.37 ± 0.05^f^
TA2	11.38 ± 0.18^a^	45.7 ± 0.08^b^	97.28 ± 0.00^a^
TA3	7.66 ± 0.16^e^	20.05 ± 0.08^e^	91.37 ± 0.05^f^
CA1	10.47 ± 0.06^c^	33.03 ± 0.08^c^	93.35 ± 0.05^e^
CA2	11.1 ± 0.06^b^	56.17 ± 0.05^a^	94.65 ± 0.00^c^
MA1	6.21 ± 0.53^f^	21.35 ± 6.76^f^	94.61 ± 0.42^d^
MA2	5.32 ± 0.00^g^	31.57 ± 0.00^d^	95.12 ± 0.06^b^

*Note*: Data are expressed as mean ± standard deviation (three replicates). ^a–g^ Different superscript letters in the same column indicate significant differences (*P* < .05).

### 3.4 Free amino acid analysis

FAAs produced by protein hydrolysis can provide the basic flavor of fermented foods (Xiao et al., 2020). It can improve the flavor of foods and further produce flavor compounds such as alcohols, aldehydes, and esters (Mi et al., 2022). The composition of free amino acids was different in different samples, and a total of 16 amino acids were detected (Tables 6 and 7). The total content of free amino acids in these seven samples was 0.257 to 0.621 mg/g (Table 6) in the middle fermentation stage and 0.220 to 0.505 mg/g in the late fermentation stage (Table 7). The increase in FAA content in the middle fermentation stage might be related to the decomposition of proteins by lactic acid bacteria, and in the late fermentation stage, some amino acids, which were also an energy and carbon source for bacterial metabolism, might be consumed by bacteria due to the limited fermentation substrate (Wang, Hou, et al., 2021; Wang, Wang, et al., 2021; Wang, Yi, et al., 2021; Ye et al., 2022). A small amount of histidine was detected in CA1 (0.009 mg/g) and CA2 (0.012 mg/g) in the late fermentation stage (Tables 6 and 7). The presence of histidine, aromatic amino acids, and sulfur‐containing amino acids in foods endows them with antioxidant properties (Tamang et al., 2021). And small amounts of threonine and serine (0.001 mg/g) were detected in TA3 in the middle fermentation stage other than the late fermentation stage, indicating that small amounts of sweet‐flavored amino acids were lost during fermentation. In the late fermentation stage, MA1 and MA2 showed a significant increase in sweet amino acids, all samples showed an increase in salty amino acids. The difference might be interpreted as follows. Different enzymes contained in the fermentation process and the different FAA metabolism rates of microorganisms led to the differences in FAA content. For example, fermented meat and rice supplemented with *Lactobacillus* and *Staphylococcus* showed an increased total FAA during fermentation (Wang, Wang, et al., 2021).

**TABLE 6:** Analysis of free amino acid composition of samples in the middle fermentation stage.

Tastes	Free amino acids (mg/g)	TA1	TA2	TA3	CA1	CA2	MA1	MA2
Savory	GLU	0.069 ± 0.003^d^	0.338 ± 0.016^a^	0.033 ± 0.002^e^	0.205 ± 0.003^c^	0.239 ± 0.007^b^	0.234 ± 0.002^b^	0.072 ± 0.001^d^
Sour	ASP	0.056 ± 0.002^b^	0.022 ± 0.001^d^	0.001 ± 0.000^f^	0.071 ± 0.001^a^	0.058 ± 0.003^b^	0.018 ± 0.000^e^	0.035 ± 0.001^c^
	Total	0.126 ± 0.005^e^	0.360 ± 0.017^a^	0.033 ± 0.002^g^	0.277 ± 0.004^c^	0.297 ± 0.011^b^	0.252 ± 0.002^d^	0.107 ± 0.003^f^
Sweet	THR	0.007 ± 0.002^d^	0.004 ± 0.000^f^	0.000^g^	0.027 ± 0.000^a^	0.022 ± 0.000^b^	0.006 ± 0.000^e^	0.010 ± 0.000^c^
Savory	SER	0.009 ± 0.000^b^	0.004 ± 0.000^e^	0.001 ± 0.000^f^	0.019 ± 0.000^a^	0.009 ± 0.000^b^	0.008 ± 0.000^c^	0.005 ± 0.000^d^
	GLY	0.011 ± 0.000^d^	0.010 ± 0.001^e^	0.019 ± 0.000^b^	0.021 ± 0.000^a^	0.014 ± 0.000^c^	0.004 ± 0.000^g^	0.008 ± 0.000^f^
	ALA	0.047 ± 0.002^c^	0.059 ± 0.003^b^	0.093 ± 0.001^a^	0.042 ± 0.001^d^	0.029 ± 0.001^f^	0.039 ± 0.000^e^	0.047 ± 0.002^c^
	Total	0.074 ± 0.004^cd^	0.077 ± 0.004^c^	0.113 ± 0.001^a^	0.108 ± 0.002^b^	0.074 ± 0.002^cd^	0.057 ± 0.001^e^	0.070 ± 0.002^d^
Bitter	HIS	ND^a^	ND^a^	ND^a^	ND^a^	ND^a^	ND^a^	ND^a^
Sweet	LYS	0.009 ± 0.004^d^	0.025 ± 0.002^b^	0.005 ± 0.001^e^	0.033 ± 0.000^a^	0.032 ± 0.000^a^	0.012 ± 0.000^c^	0.024 ± 0.000^b^
	ARG	ND^d^	0.003 ± 0.001^c^	ND^d^	0.016 ± 0.000^a^	0.006 ± 0.000^b^	0.006 ± 0.000^b^	ND^d^
	Total	0.009 ± 0.004^f^	0.027 ± 0.002^c^	0.005 ± 0.001^g^	0.049 ± 0.001^a^	0.038 ± 0.000^b^	0.019 ± 0.000^e^	0.024 ± 0.000^d^
Bitter	VAL	0.011 ± 0.000^d^	0.010 ± 0.001^e^	0.023 ± 0.000^c^	0.039 ± 0.001^a^	0.031 ± 0.001^b^	0.006 ± 0.000^f^	0.011 ± 0.001^d^
	MET	0.002 ± 0.002^d^	0.000^e^	0.005 ± 0.000^c^	0.013 ± 0.000^a^	0.010 ± 0.000^b^	0.000^e^	0.000^e^
	ILE	0.008 ± 0.004^c^	0.014 ± 0.002^b^	0.026 ± 0.005^a^	0.029 ± 0.001^a^	0.027 ± 0.003^a^	0.010 ± 0.001^bc^	0.012 ± 0.002^bc^
	LEU	0.017 ± 0.001^d^	0.012 ± 0.001^e^	0.024 ± 0.001^c^	0.052 ± 0.001^a^	0.040 ± 0.001^b^	0.005 ± 0.000^f^	0.012 ± 0.000^e^
	TYR	0.006 ± 0.000^b^	0.002 ± 0.000^c^	ND^e^	0.010 ± 0.000^a^	0.006 ± 0.000^b^	0.002 ± 0.000^c^	0.001 ± 0.000^d^
	PHE	0.020 ± 0.001^d^	0.022 ± 0.001^c^	0.022 ± 0.001^c^	0.044 ± 0.001^a^	0.034 ± 0.001^b^	0.012 ± 0.000^f^	0.018 ± 0.001^e^
	Total	0.065 ± 0.008^d^	0.060 ± 0.001^de^	0.101 ± 0.007^c^	0.186 ± 0.004^a^	0.149 ± 0.005^b^	0.035 ± 0.001^f^	0.054 ± 0.004^e^
Salty	CYS	0.001 ± 0.000^c^	0.000^d^	0.022 ± 0.000^a^	0.001 ± 0.000^cd^	0.001 ± 0.000^cd^	0.000^d^	0.002 ± 0.001^b^
	Total	0.001 ± 0.000^c^	0.000^d^	0.022 ± 0.000^a^	0.001 ± 0.000^cd^	0.001 ± 0.000^cd^	0.000^d^	0.002 ± 0.001^b^
TAA	(Total Amino Acids)	0.275 ± 0.020^e^	0.525 ± 0.021^c^	0.273 ± 0.008^e^	0.621 ± 0.010^a^	0.557 ± 0.018^b^	0.362 ± 0.003^d^	0.257 ± 0.009^e^

*Note*: Data are expressed as mean ± standard deviation (three replicates). ^a–g^Different superscript letters in the same column indicate significant differences (*p* < .05).

Abbreviation: ND, not detected.

**TABLE 7:** Analysis of free amino acid composition of samples in the late fermentation stage.

Tastes	Free amino acids (mg/g)	TA1	TA2	TA3	CA1	CA2	MA1	MA2
Savory	GLU	0.047 ± 0.002^f^	0.294 ± 0.005^a^	0.025 ± 0.000^f^	0.157 ± 0.003^d^	0.219 ± 0.044^c^	0.263 ± 0.012^b^	0.088 ± 0.000^e^
Sour	ASP	0.047 ± 0.002^c^	0.032 ± 0.001^d^	0.006 ± 0.001^e^	0.059 ± 0.002^b^	0.068 ± 0.011^a^	0.011 ± 0.003^e^	0.003 ± 0.000^e^
	Total	0.094 ± 0.005^d^	0.327 ± 0.004^a^	0.031 ± 0.001^e^	0.216 ± 0.005^c^	0.287 ± 0.055^b^	0.274 ± 0.015^b^	0.091 ± 0.000^d^
Sweet	THR	0.047 ± 0.002^a^	0.008 ± 0.001^d^	ND^f^	0.025 ± 0.000^b^	0.021 ± 0.002^c^	0.007 ± 0.001^d^	0.003 ± 0.000^e^
Savory	SER	0.047 ± 0.002^a^	0.007 ± 0.000^d^	ND^f^	0.014 ± 0.000^b^	0.007 ± 0.001^d^	0.010 ± 0.000^c^	0.005 ± 0.000^e^
	GLY	0.047 ± 0.002^a^	0.010 ± 0.001^d^	0.015 ± 0.000^c^	0.017 ± 0.001^b^	0.017 ± 0.001^b^	0.008 ± 0.000^e^	0.016 ± 0.000^bc^
	ALA	0.047 ± 0.002^d^	0.048 ± 0.001^d^	0.068 ± 0.000^c^	0.031 ± 0.000^e^	0.031 ± 0.000^e^	0.072 ± 0.003^b^	0.098 ± 0.001^a^
	Total	0.187 ± 0.009^a^	0.074 ± 0.002^f^	0.083 ± 0.001^de^	0.087 ± 0.002^d^	0.077 ± 0.002^ef^	0.097 ± 0.003^c^	0.122 ± 0.001^b^
Bitter	HIS	ND^c^	ND^c^	0.012 ± 0.000^a^	0.009 ± 0.000^b^	ND^c^	ND^c^	ND^c^
Sweet	LYS	0.026 ± 0.002^ab^	0.013 ± 0.001^cd^	0.005 ± 0.000^d^	0.018 ± 0.001^bc^	0.029 ± 0.014^a^	0.017 ± 0.001b^c^	0.005 ± 0.001^d^
	ARG	NDe	0.003 ± 0.000^d^	ND^e^	0.014 ± 0.001^a^	0.008 ± 0.002^b^	0.006 ± 0.000^c^	ND^e^
	Total	0.026 ± 0.002^b^	0.017 ± 0.000^b^	0.017 ± 0.000^b^	0.041 ± 0.002^a^	0.037 ± 0.016^a^	0.023 ± 0.001^b^	0.005 ± 0.001^ca^
Bitter	VAL	0.047 ± 0.002^a^	0.008 ± 0.002^c^	0.024 ± 0.000b	0.030 ± 0.012^b^	0.029 ± 0.005^b^	0.012 ± 0.001^c^	0.027 ± 0.006^b^
	MET	0.047 ± 0.002^a^	0.007 ± 0.000^cd^	0.005 ± 0.000^de^	0.014 ± 0.002^b^	0.010 ± 0.003^c^	0.002 ± 0.002^e^	0.007 ± 0.000^cd^
	ILE	0.047 ± 0.002^a^	0.019 ± 0.007^d^	0.021 ± 0.001^cd^	0.035 ± 0.001^b^	0.026 ± 0.004^c^	0.007 ± 0.004^e^	0.019 ± 0.001^d^
	LEU	0.047 ± 0.002^a^	0.001 ± 0.000^c^	0.000^c^	0.018 ± 0.017^bc^	0.019 ± 0.019^b^	0.012 ± 0.001^bc^	0.021 ± 0.001^b^
	TYR	ND^c^	ND^c^	ND^c^	0.007 ± 0.001^b^	0.009 ± 0.000^a^	ND^c^	ND^c^
	PHE	0.026 ± 0.002^c^	0.024 ± 0.001^c^	0.024 ± 0.000^c^	0.040 ± 0.002^a^	0.03 ± 0.004^b^	0.017 ± 0.001^d^	0.024 ± 0.000^c^
	Total	0.214 ± 0.011^a^	0.059 ± 0.008^e^	0.074 ± 0.002^de^	0.143 ± 0.024b	0.123 ± 0.028^bc^	0.051 ± 0.008^e^	0.098 ± 0.006^cd^
Salty	CYS	0.047 ± 0.002^a^	0.046 ± 0.000^a^	0.046 ± 0.000^a^	0.046 ± 0.000^a^	0.046 ± 0.000^a^	0.046 ± 0.000^a^	0.046 ± 0.000^a^
	Total	0.047 ± 0.002^a^	0.001 ± 0.001^c^	0.021 ± 0.004^b^	0.002 ± 0.001^c^	0.001 ± 0.001^c^	0.001 ± 0.000^c^	0.019 ± 0.000^b^
TAA	(Total Amino Acids)	0.265 ± 0.008^bc^	0.478 ± 0.010^a^	0.220 ± 0.001^c^	0.490 ± 0.033^a^	0.505 ± 0.119^a^	0.445 ± 0.026^a^	0.330 ± 0.002^b^

*Note*: Data are expressed as mean ± standard deviation (three replicates). ^a–g^Different superscript letters in the same column indicate significant differences (*p* < .05).

Abbreviation: ND, not detected.

### 3.5 Microbial community characteristics

#### 3.5.1 Microbial diversity sequencing analysis

**FIGURE 3:**
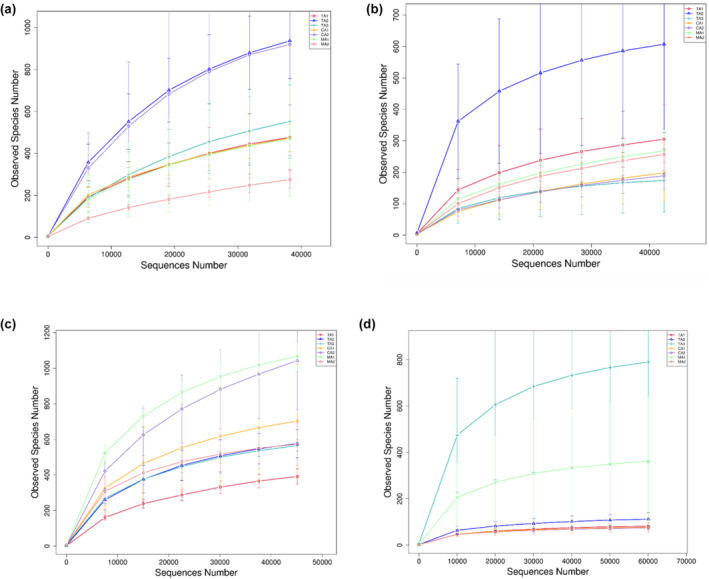
Rarefaction curves of sequencing reads of (a) bacterial 16S rDNA gene and (b) fungal ITS rDNA gene from the samples in the middle fermentation stage and (c) bacterial 16S rDNA gene and (d) fungal ITS rDNA gene from the samples in the late fermentation stage.

In the middle fermentation stage, the quantity of observed species of bacteria ranged from 275 (MA2) to 937 (TA2) (Table 8), and the quantity of observed species of fungi ranged from 174 (TA3) to 607 (TA2) (Table 9), indicating that the diversity of bacterial flora was higher than that of fungi in all samples and that bacteria were the most important microbial community in the fermentation process. These microbial strains were required for the formation of unique flavors, aromas, and textures (Ilango & Antony, 2021). In the late fermentation stage, the quantity of observed bacterial species ranged from 391 (TA1) to 1067 (MA1) (Table 8), and the quantity of observed fungal species ranged from 75 (CA2) to 790 (TA3) (Table 9). In addition, when the sequencing depth reached 4 × 10^4^ reads, the rarefaction curves of the sequencing reads of the samples in both middle and late fermentation were smooth (Figure 3), indicating that the number of OUTs in each sample was close to saturation. In other words, the sequencing data were sufficient and reasonable.

**TABLE 8:** Quantities of observed species, community richness index (Chao 1 and Ace), community diversity index (Shannon and Simpson), and estimated sample good's coverage for 16S rRNA analysis of the samples in the middle and late fermentation stages.

Samples	Observed species	Chao 1	Ace	Shannon	Simpson	Good's coverage
Middle fermentation						
TA1	477	611.526	668.442	4.011	0.885	0.995
TA2	937	1080.001	1147.308	4.197	0.832	0.992
TA3	553	716.159	816.714	3.453	0.797	0.994
CA1	475	746.650	756.635	3.892	0.843	0.995
CA2	920	1043.451	1141.799	3.843	0.808	0.994
MA1	471	678.377	754.404	3.347	0.805	0.995
MA2	275	552.809	662.756	2.442	0.706	0.996
Late fermentation						
TA1	391	559.275	570.194	1.114	0.210	0.996
TA2	576	698.807	717.102	1.627	0.289	0.997
TA3	565	651.079	691.153	3.827	0.871	0.997
CA1	703	865.770	898.137	4.271	0.866	0.995
CA2	1042	1443.785	1506.734	2.262	0.414	0.991
MA1	1067	1201.065	1247.389	2.858	0.444	0.995
MA2	573	644.674	658.250	4.001	0.850	0.997

**TABLE 9:** Quantities of observed species, community richness index (Chao 1 and Ace), community diversity index (Shannon and Simpson), and estimated sample good's coverage for ITS rRNA analysis of the samples in the middle and late fermentation stages.

Samples	Observed species	Chao 1	Ace	Shannon	Simpson	Good's coverage
Middle fermentation						
TA1	305	376.825	407.273	2.947	0.742	0.998
TA2	607	702.363	712.190	4.596	0.775	0.997
TA3	174	188.840	198.277	1.289	0.447	0.999
CA1	198	320.186	363.604	2.052	0.659	0.998
CA2	188	284.163	306.164	2.106	0.550	0.998
MA1	268	369.202	407.262	2.499	0.675	0.997
MA2	256	372.354	395.507	2.095	0.586	0.997
Late fermentation						
TA1	82	87.118	89.629	0.628	0.139	1.000
TA2	112	127.299	126.495	1.404	0.386	1.000
TA3	790	840.988	857.307	5.447	0.891	0.998
CA1	77	82.321	85.281	0.819	0.188	1.000
CA2	75	81.333	85.362	2.258	0.752	1.000
MA1	362	393.269	399.361	2.957	0.658	0.999
MA2	76	83.469	86.720	1.584	0.551	1.000

#### 3.5.2 Structure of bacterial flora

**FIGURE 4:**
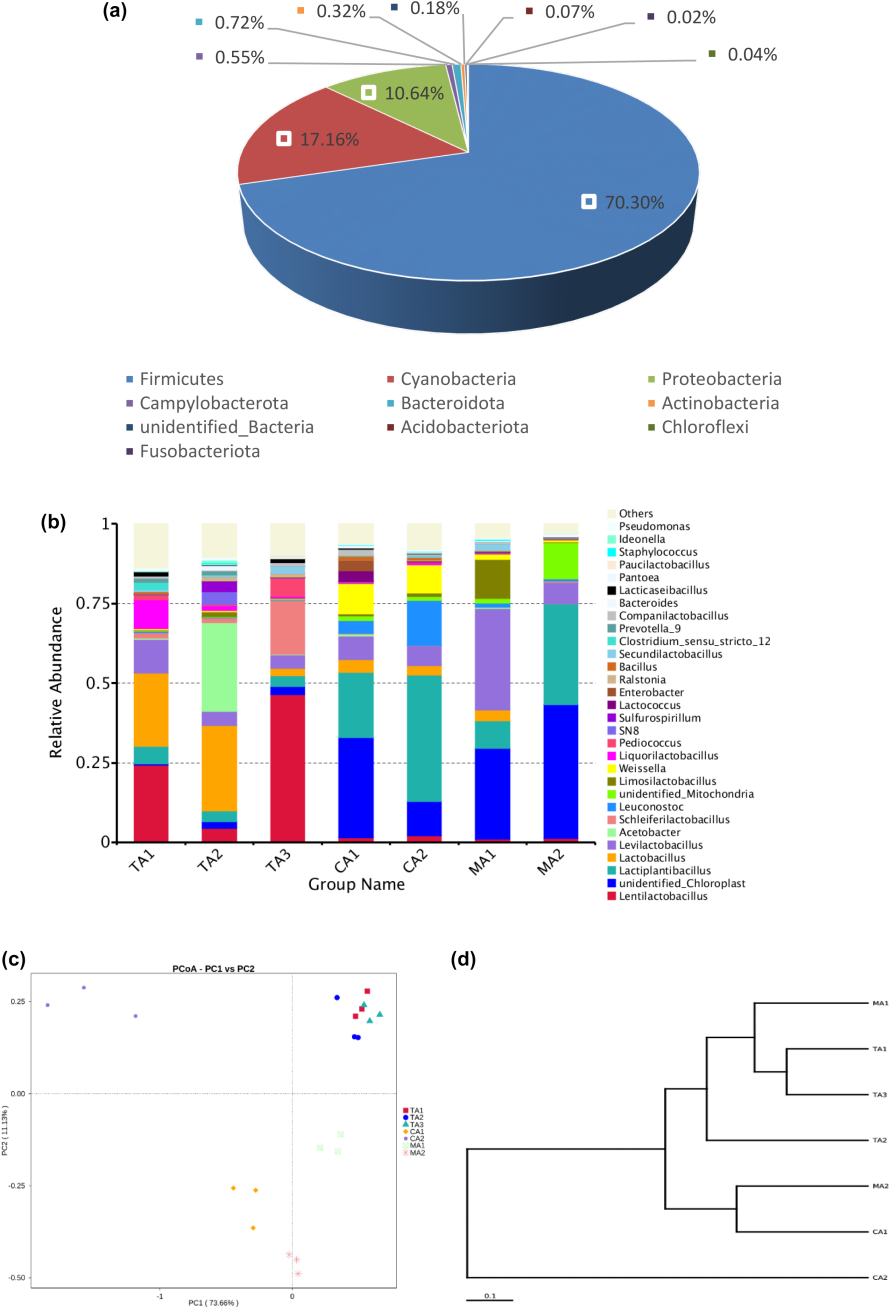
Relative abundances of bacterial 16S rRNA genes from the samples in the middle fermentation stage at (a) phylum level and (b) genus level. Community comparison of bacterial microbiota in the samples: (c) principal coordinates analysis of bacteria (PCoA) and (d) weighted pair group method with arithmetic mean (UPGMA) based on beta diversity distance of bacteria in the samples in the middle fermentation stage.

In the middle fermentation stage, five phyla were the predominant phyla in the samples of tomato acid, chili acid, and mixed acid (Figure 4a): *Firmicutes* (70.30%), *Cyanobacteria* (17.16%), *Proteobacteria* (10.64%), *Bacteroidota* (0.72%), and *Campylobacterota* (0.55%). Several bacterial genera were detected in seven samples (Figure 4b), and the genera with high abundances included *Lentilactobacillus*, *Lactiplantibacillus*, *Lactobacillus*, and *Levilactobacillus*. *Lentilactobacillus* and *Lactiplantibacillus* were detected in all samples. The abundance of *Lactiplantibacillus* was higher in the chili acid and mixed acid samples, probably due to differences in the raw materials of the samples. *Lactiplantibacillus* played an active role in the preparation of sour soup because it could switch to the phosphoketolase pathway in sugar metabolism so as to produce more diverse metabolites depending on the environmental conditions (Nsogning Dongmo et al., 2016). The dominant genera of the three tomato acid samples were similar, which were mainly *Lentilactobacillus* and *Lactobacillus*. The dominant genera in CA1 were mainly *Lactiplantibacillus* and *Levilactobacillus*, which were the same as those in the mixed acid samples (MA1 and MA2). The dominant genera in CA2 were *Lactiplantibacillus* and *Leuconostoc*. *Leuconostoc* was reported to be one of the major lactic acid bacteria in spontaneously fermented foods (Xie et al., 2019). *Leuconostoc* could produce organic acids, carbon dioxide, and other substances that caused a significant decrease in pH and played an important role in the initiation of fermentation and the formation of product flavor in the early fermentation stage (Wang et al., 2020). In summary, *Lactiplantibacillus* and *Lentilactobacillus* were the dominant bacterial genera in the samples in the middle fermentation stage, similar to the results of previous studies (Lin et al., 2020; Xiong et al., 2021). The use of *Lactiplantibacillus* in making fresh fermented rice flour effectively inhibited the growth of pathogenic bacteria and shortened the fermentation period (Wang, Hou, et al., 2021; Wang, Wang, et al., 2021; Wang, Yi, et al., 2021). *Lactiplantibacillus* was often used in food production to improve the texture, aroma, and taste of foods and also acted as a probiotic to improve human and animal health. In addition, *Lactiplantibacillus* is considered as one of the major agricultural bacteria to ensure plant health and is often used as a biocide and biocontrol agent (Mota‐Gutierrez & Cocolin, 2021). Three tomato acid samples were clustered together (Figure 4c), indicating that the difference in bacterial flora structure between them was small. In addition, the results of the UPGMA analysis were largely similar to the PCoA results in that the tomato acid samples were clustered together and had similar bacterial flora (Figure 4d).

**FIGURE 5:**
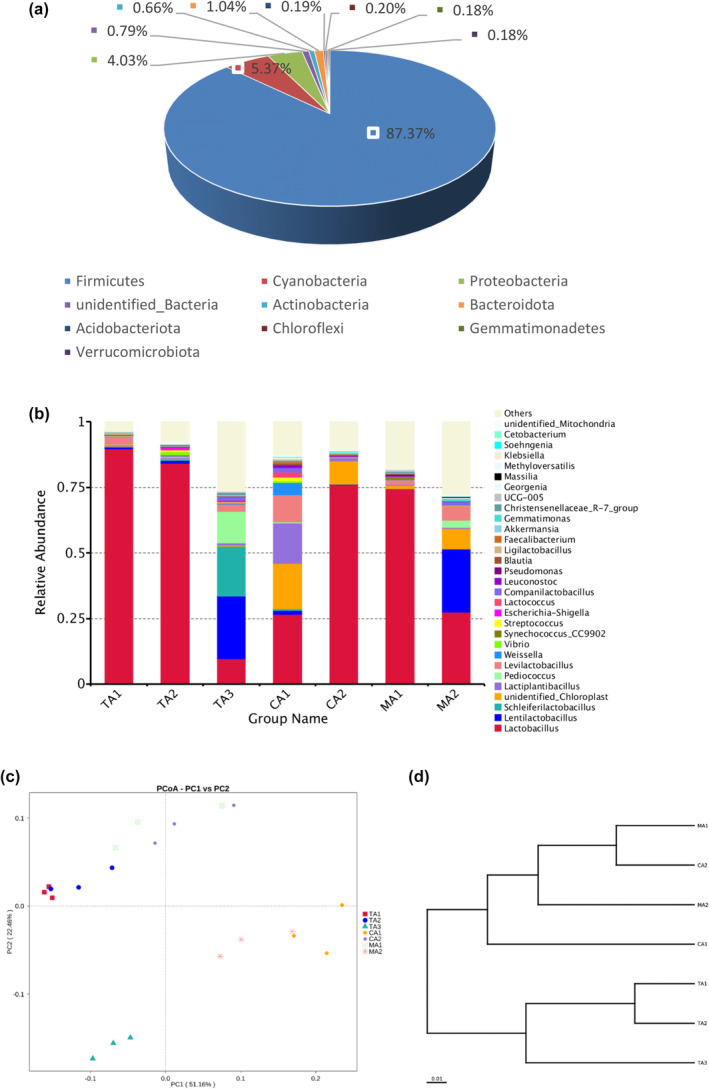
Relative abundances of bacterial 16S rRNA genes from the samples in the late fermentation stage at (a) phylum level and (b) genus level. Community comparison of bacterial microbiota in the samples: (c) principal coordinates analysis of bacteria (PCoA) and (d) weighted pair group method with arithmetic mean (UPGMA) based on beta diversity distance of bacteria in the samples in the late fermentation stage.

In the late fermentation stage, the abundances of *Firmicutes* (87.37%) and *Proteovacteria* (4.03%) were significantly increased, the abundance of *Actinobacteria* (0.66%) was slightly increased; and the abundance of *Cyanobacteria* (5.37%) was significantly decreased (Figure 5a). The abundance of *Lactobacillus* increased significantly in all samples and became the dominant genus in all samples except TA3, where the dominant genus was *Lentilactobacillus* (Figure 5b). Obviously, the abundances of *Lactiplantibacillus* and *Lentilactobacillus*, which were slightly dominant in the middle fermentation stage, decreased, indicating that they might play a main role in the early stage. The abundances of other bacteria also gradually decreased during fermentation because they were at a competitive disadvantage in the late stage and were inhibited by various advantageous bacteria competing for nutrients or their metabolites (Xie et al., 2020). In conclusion, the dominant genus of bacteria in red sour soup in the late fermentation stage was mainly *Lactobacillus*, similar to the previous study (Lin et al., 2022). *Lactobacillus* is a Gram‐positive bacterium that produces lactic acid to create an acidic environment and can inhibit the growth of other bacteria and a variety of pathogens and prevent the softening of food materials (Nie et al., 2022). In addition, *Lactobacillus* is the major contributor to the ripening of fermented vegetables, and *Lactobacillus* and other dominant genera can convert carbohydrates in the fermented substrate into energy to accelerate the fermentation process (Guan et al., 2022). In addition, TA1 and TA2 were clustered closely (Figure 5c). In other words, their bacterial flora structures were similar. TA3 was close to the PC1 axis, as found in the UPGMA analysis results of bacteria (Figure 5d).

These results indicated that the composition of the bacterial community changed considerably during fermentation and tended to decrease in diversity, but the genus *Lactobacillus* always dominated the bacterial community and played an important role in the fermentation of red sour soup. LAB, including *Lactobacillus*, *Lactococcus lactis*, *Weissella*, and *Leuconostoc*, are the key microbiota for the acidic fermentation of vegetables (Guan et al., 2020). In China, fermented foods dominate the food processing industry and food market, and lactic acid flora is the most important microorganism in the food industry (Leite De Souza, 2021). Several studies reported that LAB secreted a variety of acids and antimicrobial substances to inhibit the growth of pathogenic microorganisms and improve food safety and had perfect antioxidant activity, antimicrobial activity, and anti‐inflammatory activity (Lin et al., 2022; Rodzi & Lee, 2021).

#### 3.5.3 Structure of fungal flora

**FIGURE 6:**
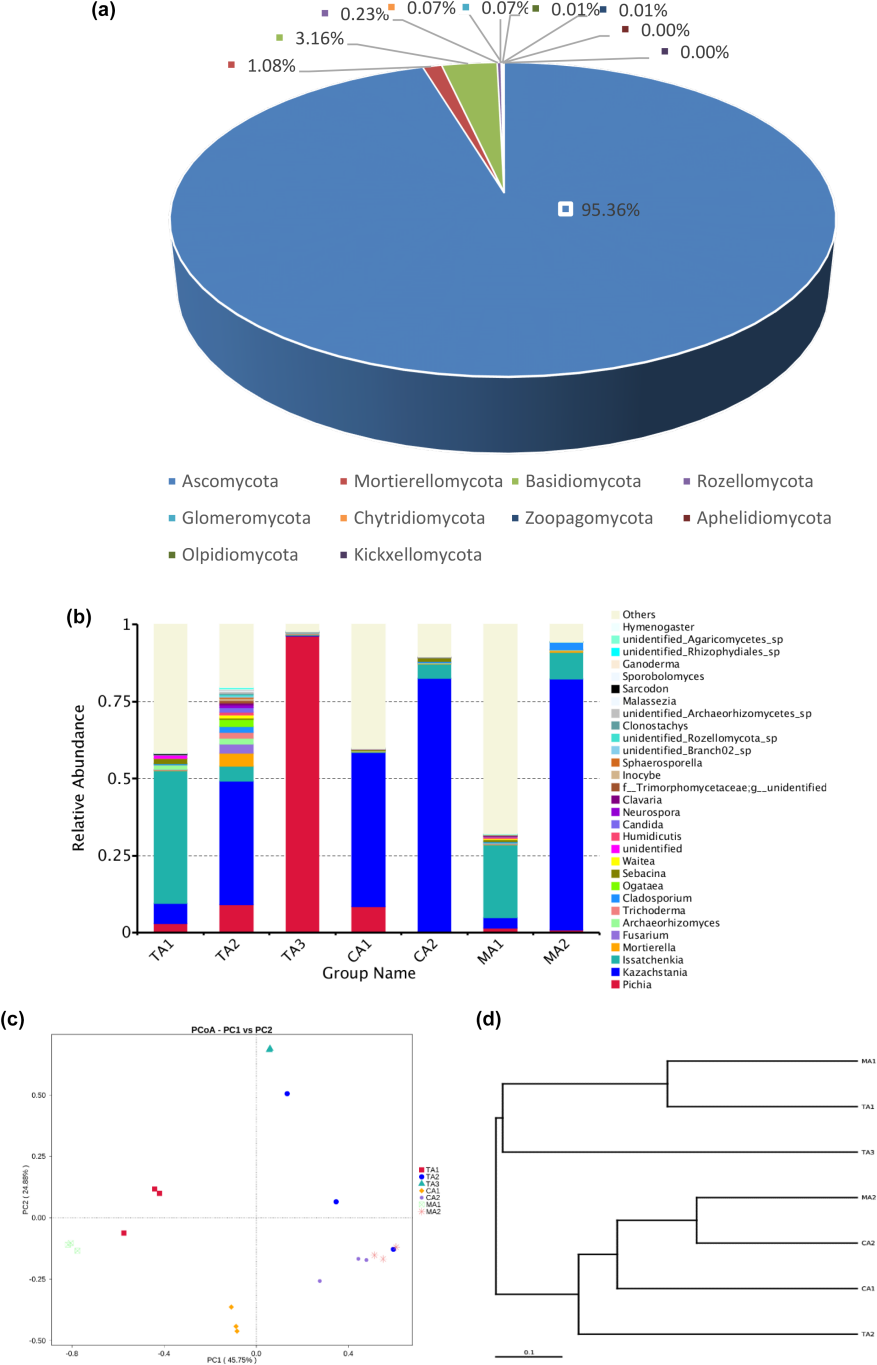
Relative abundances of fungal ITS rRNA genes from the samples in the middle fermentation stage at (a) phylum level and (b) genus level. Community comparison of fungal microbiota in the samples: (c) principal coordinates analysis of fungi (PCoA) and (d) weighted pair group method with arithmetic mean (UPGMA) based on beta diversity distance of fungi in the samples in the middle fermentation stage.

In the middle fermentation stage, the fungi in the samples of tomato acid, chili acid, and mixed acid mainly included four phyla (Figure 6a): *Ascomycota* (95.36%), *Basidiomycota* (3.16%), *Mortierellomycota* (1.08%), and *Rozellomycota* (0.23%). Several fungal genera were detected in seven samples (Figure 6b) and the dominant genera included *Pichia*, *Kazachstania*, *Issatchenkia*, and *Mortierella*. *Mortierella* are root microorganisms of various plants and may be carried by the raw materials of red sour soup (Wang et al., 2020). Both *Pichia* and *Kazachstania* were detected in all the samples. *Pichia* has good probiotic potential to enhance the contents of folate and phenolic acid in fermented cereal foods (Liu et al., 2020). However, the higher abundance of *Pichia* in tomato acid samples was different from the previous report that the higher abundance of *B. bruxellensisis* was found in tomato acid (Lin et al., 2020), due to the difference in raw materials, production parameters, fermentation temperature, and fermentation environment. TA2 had the highest fungal diversity and TA3 had the lowest fungal diversity (Figure 6b). *Pichia* was the main fungal genus in TA3 and *Kazachstania* was the main fungal genus in TA2, CA1, CA2, and MA2. *Kazachstania* and *Pichia* were reported to be the main yeast genera in fermented vegetables (Zhao et al., 2022). In the PCoA results of fungi (Figure 6c), CA1, CA2, and MA2 were close to the PC1 axis. In addition, the fungal clustering results of the UPGMA samples were basically the same as the PCoA results (Figure 6d).

**FIGURE 7:**
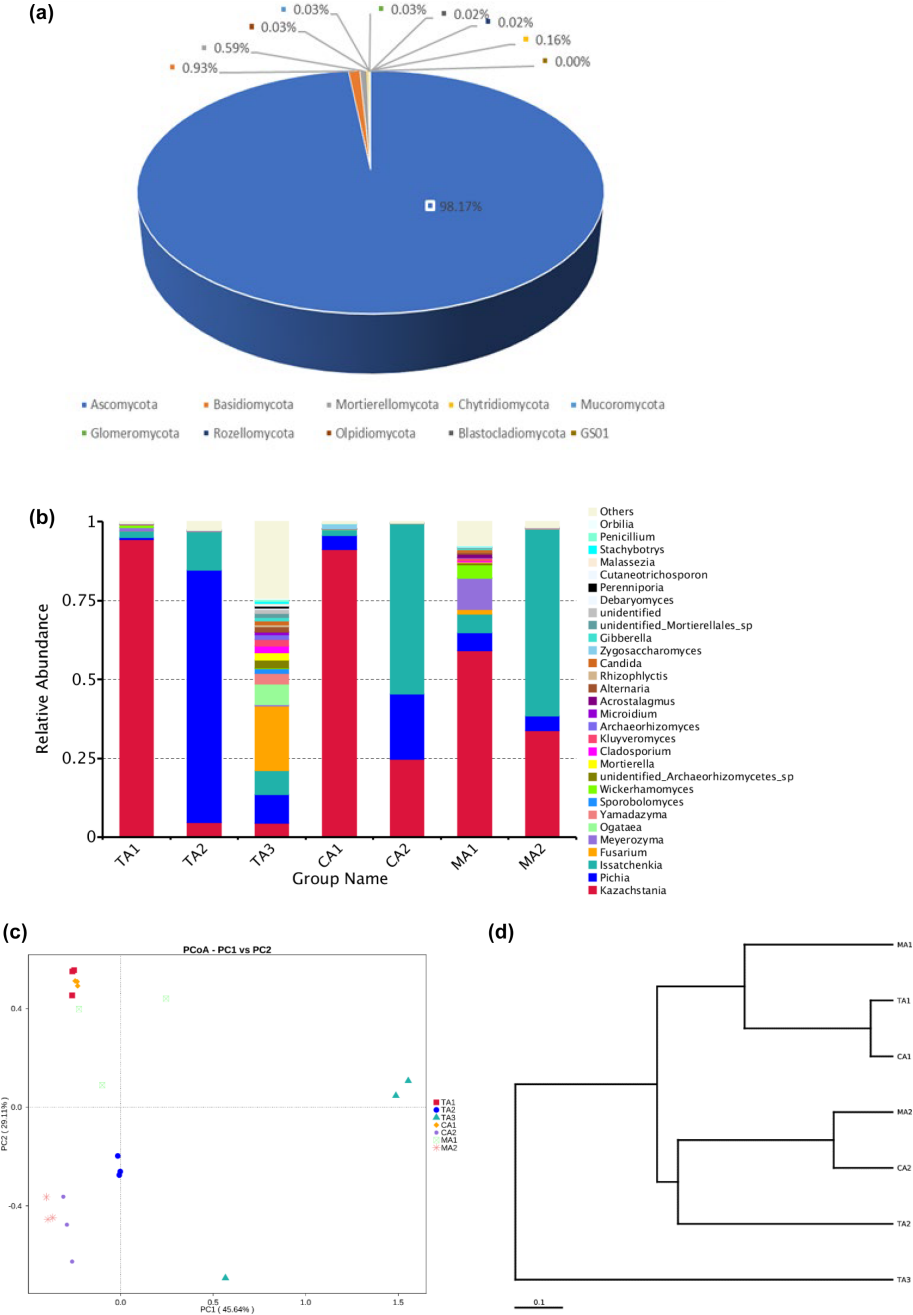
Relative abundances of fungal ITS rRNA genes from the samples in the late fermentation stage at (a) phylum level and (b) genus level. Community comparison of fungal microbiota in the samples: (c) principal coordinates analysis of fungi (PCoA) and (d) weighted pair group method with arithmetic mean (UPGMA) based on beta diversity distance of fungi in the samples in the late fermentation stage.

In the late fermentation stage, the abundance of *Ascomycota* (98.17%) was significantly higher (Figure 7a). Several fungal genera were detected in seven samples (Figure 7b) and the dominant genera included *Kazachstania*, *Pichia*, *Issatchenkia*, and *Fusarium*. The absolutely dominant genus (*Pichia*) in TA3 in the middle fermentation stage lost their dominant positions in the late fermentation stage (Figure 7b), and many other genera appeared. *Fusarium* became the dominant genus, indicating that TA3 started to spoil and thus led to its lowest sensory evaluation score. Other samples were dominated by yeasts, and the abundance of miscellaneous bacteria was low. Other genera were at a competitive disadvantage in the late fermentation stage. The change in the flora might be interpreted as follows. A large amount of *Pichia* in the middle fermentation stage provided nutrients to the miscellaneous bacteria (Zang et al., 2018). In addition, the fungal flora in TA3 was significantly different from that in other samples (Figure 7c), as found in the results of the UPGMA analysis of fungi (Figure 7d). The difference might be attributed to the presence of pathogenic bacteria in the raw material of TA3.

Our results showed that most of the fungi found in various components of split‐fermented red sour soup belonged to *Ascomycota* and *Basidiomycota*, as reported in previous studies on other types of traditional Chinese fermented vegetables (Guan et al., 2020; Lin et al., 2020). Interestingly, *Kazachstania* always dominated the fungal community and played an important role in the fermentation of different parts of red sour soup in the middle and late fermentation stages. All samples contained a large amount of yeast, indicating that they played an important role in the fermentation process. Yeasts might provide carbon and nitrogen sources for fermentation and promote the growth of other microorganisms that contribute to the taste and flavor of fermented vegetables. Yeasts were considered as the main fungi affecting the formation of flavor in many fermentation processes (Sakandar et al., 2020; Zang et al., 2018). In a word, the microbial community showed no significant change during the fermentation period, but the relative abundance of microbial communities experienced significant dynamic changes in different stages due to microbial competition, substrate limitation, and the fermentation environment. The further work will screen out the strains with excellent fermentation characteristics in spilt‐fermented red sour soup and mix‐fermented red sour soup and develop suitable fermentation starters for red sour soup.

## 4 CONCLUSIONS

In this study, we compared the quality characteristics and microbial communities in different components of split‐fermented red sour soup in two fermentation stages so as to understand the quality, flavor, and microbial community during the fermentation of red sour soup. The titratable acidity of mixed acids was lower than that of tomato acid and chili acid in the middle stage. The cell viability of lactic acid bacteria was mostly higher than that of yeasts during the whole fermentation. The signal intensity of sour and sensory increased and bitter and salty decreased in the late stage compared with the middle stage. The total free amino acid content of the samples was distributed in the range of 0.257 ± 0.009–0.648 ± 0.010 mg/g at the middle stage. In addition, the antioxidant capacity of the samples was different between the two stages, the value of ABTS free radical scavenging capacity was more than 91.37 ± 0.05%. *Lactiplantibacillus* and *Lentilactobacillus* were the dominant bacterial genera in the samples in the middle stage, *Lactobacillus* was dominant in the late stage, and *Pichia* and *Kazachstania* were detected in all the samples in two stages. This study improved the understanding of the quality characteristics and microbial communities of traditional split‐fermented red sour soup.

## AUTHOR CONTRIBUTIONS

**Na Liu:** Data curation (equal); formal analysis (equal); funding acquisition (equal); investigation (equal); methodology (equal); visualization (equal); writing – original draft (equal). **Yue Hu:** Data curation (equal); formal analysis (equal); investigation (equal); methodology (equal); software (equal); visualization (equal); writing – original draft (equal). **Mingxia Wu:** Formal analysis (equal); visualization (equal). **Likang Qin:** Conceptualization (equal); funding acquisition (equal); investigation (equal); project administration (equal); supervision (equal); writing – review and editing (equal). **Aiming Bao:** Resources (equal). **Weijun Qin:** Resources (equal). **Song Miao:** Conceptualization (equal); project administration (equal); supervision (equal); writing – review and editing (equal).

## CONFLICT OF INTEREST STATEMENT

The authors declare that they do not have any conflict of interest.

## ETHICS STATEMENT

Written informed consent was obtained from all study participants.

## Data Availability

Data available on request from the authors.
